# Erioflorin Stabilizes the Tumor Suppressor Pdcd4 by Inhibiting Its Interaction with the E3-ligase β-TrCP1

**DOI:** 10.1371/journal.pone.0046567

**Published:** 2012-10-02

**Authors:** Johanna S. Blees, Heidi R. Bokesch, Daniela Rübsamen, Kathrin Schulz, Larissa Milke, Magdalena M. Bajer, Kirk R. Gustafson, Curtis J. Henrich, James B. McMahon, Nancy H. Colburn, Tobias Schmid, Bernhard Brüne

**Affiliations:** 1 Institute of Biochemistry I, Faculty of Medicine, Goethe-University Frankfurt, Frankfurt, Germany; 2 Molecular Targets Laboratory, Center for Cancer Research, National Cancer Institute, Frederick National Laboratory for Cancer Research, Frederick, Maryland, United States of America; 3 SAIC-Frederick, Inc., Frederick National Laboratory for Cancer Research, Frederick, Maryland, United States of America; 4 Laboratory of Cancer Prevention, Center for Cancer Research, National Cancer Institute, Frederick National Laboratory for Cancer Research, Frederick, Maryland, United States of America; Christian-Albrechts-University Kiel, Germany

## Abstract

Loss of the tumor suppressor Pdcd4 was reported for various tumor entities and proposed as a prognostic marker in tumorigenesis. We previously characterized decreased Pdcd4 protein stability in response to mitogenic stimuli, which resulted from p70^S6K1^-dependent protein phosphorylation, β-TrCP1-mediated ubiquitination, and proteasomal destruction. Following high-throughput screening of natural product extract libraries using a luciferase-based reporter assay to monitor phosphorylation-dependent proteasomal degradation of the tumor suppressor Pdcd4, we succeeded in showing that a crude extract from *Eriophyllum lanatum* stabilized Pdcd4 from TPA-induced degradation. Erioflorin was identified as the active component and inhibited not only degradation of the Pdcd4-luciferase-based reporter but also of endogenous Pdcd4 at low micromolar concentrations. Mechanistically, erioflorin interfered with the interaction between the E3-ubiquitin ligase β-TrCP1 and Pdcd4 in cell culture and in *in vitro* binding assays, consequently decreasing ubiquitination and degradation of Pdcd4. Interestingly, while erioflorin stabilized additional β-TrCP-targets (such as IκBα and β-catenin), it did not prevent the degradation of targets of other E3-ubiquitin ligases such as p21 (a Skp2-target) and HIF-1α (a pVHL-target), implying selectivity for β-TrCP. Moreover, erioflorin inhibited the tumor-associated activity of known Pdcd4- and IκBα-regulated αtranscription factors, that is, AP-1 and NF-κB, altered cell cycle progression and suppressed proliferation of various cancer cell lines. Our studies succeeded in identifying erioflorin as a novel Pdcd4 stabilizer that inhibits the interaction of Pdcd4 with the E3-ubiquitin ligase β-TrCP1. Inhibition of E3-ligase/target-protein interactions may offer the possibility to target degradation of specific proteins only as compared to general proteasome inhibition.

## Introduction

Programmed cell death 4 (Pdcd4) is a novel tumor suppressor that inhibits translation rather than transcription. Specifically, Pdcd4 interferes with the activity of the eukaryotic initiation factor (eIF) 4A by displacing the scaffold protein eIF4G from its binding to the RNA helicase eIF4A [1]. As a consequence, Pdcd4 attenuates neoplastic transformation, AP-1 transactivation, intravasation, and invasion *in vitro* [2], [3]. In addition, Pdcd4-deficient mice were shown to be more susceptible to the two stage skin carcinogenesis model, whereas transgenic overexpression of Pdcd4 decreased papilloma incidence and multiplicity [4], [5]. In line, Pdcd4 is lost in various tumor entities such as lung, colon, breast, ovarian and pancreatic cancer [6], [7]. Interestingly, loss of Pdcd4 appears not to be attributable to mutational inactivation [8]. Instead, post-transcriptional regulatory mechanisms appear to control Pdcd4 expression in tumors. Specifically, in addition to miR-21-dependent repression of Pdcd4 expression [9], [10], increased proteasomal degradation was recently identified to determine Pdcd4 levels in response to mitogens and inflammatory tumor environments [5], [11], [12]. Mechanistically, Pdcd4 protein contains a p70^S6K1^ consensus phosphorylation sequence directly followed by the binding motif for the E3-ubiquitin ligase β-transducin repeat-containing protein (β-TrCP). Activation of p70^S6K1^ in response to mitogens such as the phorbol ester 12-*O*-tetradecanoylphorbol-13-acetate (TPA) results in phosphorylation of Pdcd4, followed by binding of β-TrCP, polyubiquitination and subsequent proteasomal degradation [5], [11]. Overactivation of the PI3K-Akt-mTOR-p70^S6K^ axis is common in many tumor types [13]. Consequently, interference with this signaling cascade is widely used for current tumor therapeutic regimens, e.g. mTOR inhibitors are in clinical use for the treatment of renal cell carcinomas and mantle-cell lymphomas [14].

The E3-ubiquitin ligase β-TrCP represents the substrate recognition subunit of the SCF (Skp1-Cul1-FBP) ligase complex that transfers ubiquitin molecules to label target-proteins for proteasomal degradation [15]. Despite the high diversity in reported β-TrCP-targets, including tumor suppressive (e.g. IκBα) and oncogenic factors (e.g. β-catenin), β-TrCP is considered an oncoprotein [16]. In line, cancer tissues are often associated with elevated β-TrCP levels [17]. Furthermore, β-TrCP-deficient cells were shown to be more sensitive to various anti-cancer drugs such as doxorubicin, tamoxifen and paclitaxel [18]. Targeting the ubiquitin proteasome system (UPS) for tumor therapeutic purposes was proven to be a promising approach by the introduction of the general proteasome inhibitor velcade (bortezomib) for the treatment of multiple myeloma [19]. As expected, the unselective inhibition of protein degradation causes adverse side effects, limiting the use of such an approach. Since substrate specificity of the UPS is achieved by the E3-ligases, this class of proteins offers a novel avenue for tumor therapies. To date, the only compounds targeting an E3-ubiquitin ligase in clinical trials are substances of the nutlin family that disrupt the Hdm2-p53 binding and, thus prevent Hdm2-induced p53 degradation [20]. Accordingly, attenuating the interaction of β-TrCP with its target-proteins could be a promising approach for the development of proteasomal degradation targeting drugs for tumor therapies. Stabilization of the β-TrCP-target Pdcd4 provides an attractive tool for the identification of novel β-TrCP-inhibitors, which might be further developed for use in anti-tumor therapies.

**Figure 1:**
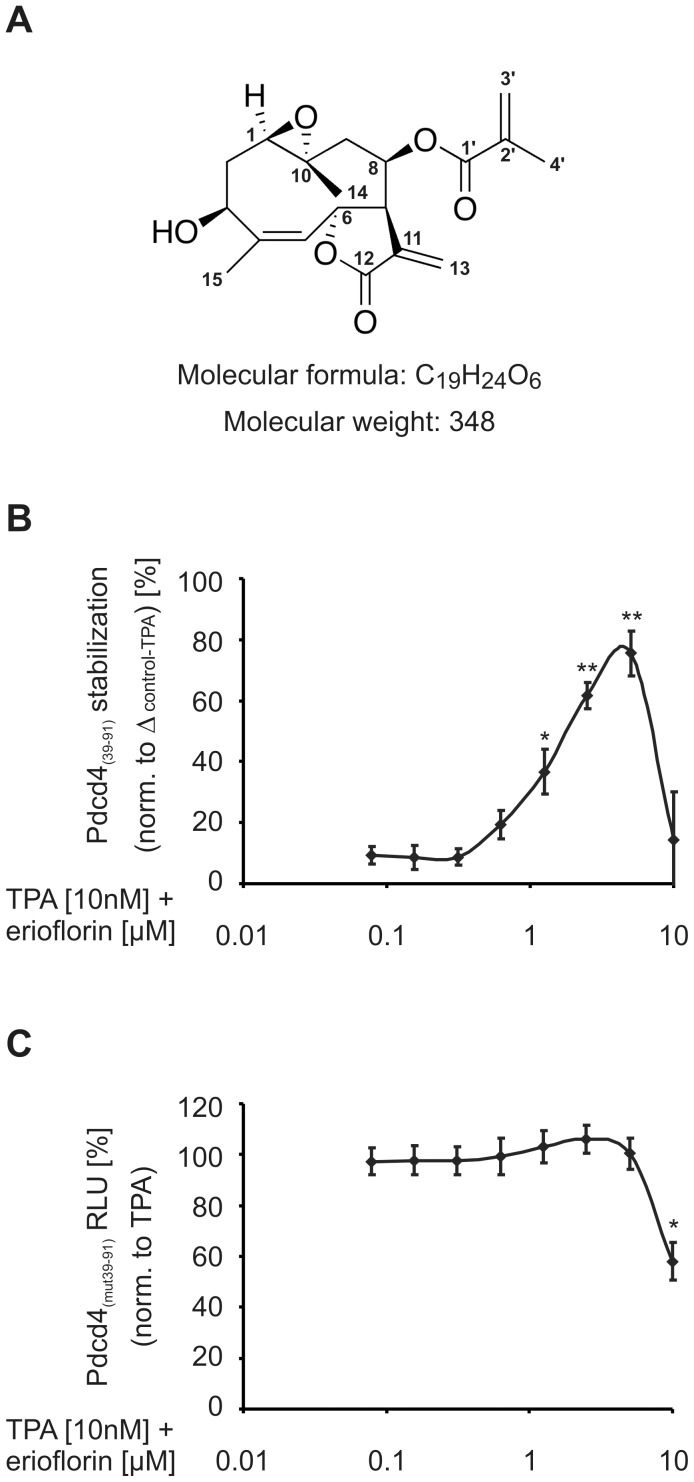
Erioflorin stabilizes Pdcd4_(39–91)_luc from TPA-induced degradation. (A) Structure of erioflorin (Mwt = 348.4). (B) Stably Pdcd4_(39–91)_luc expressing HEK293 cells were treated for 8 h with TPA (10 nM) with increasing concentrations of erioflorin (0.0625–10 µM). Pdcd4 stabilizing activity was determined relative to Δ(RLU_control_–RLU_TPA-only_). (C) Stably Pdcd4_(mut39–91)_luc expressing HEK293 cells were treated as in (B). Luciferase activity is given relative to TPA-treated controls. All data are presented as means ± SEM (n≥3, *p<0.05, **p<0.01).

Therefore, we set out to identify novel stabilizers of the tumor suppressor Pdcd4, which interfere specifically with β-TrCP-mediated degradation of Pdcd4.

## Materials and Methods

### Materials

All chemicals were purchased from Sigma-Aldrich unless noted otherwise. Rapamycin and TPA (12-O-tetradecanolyphorbol-13-acetate) were from LC Laboratories. Anti-Pdcd4, anti-phospho-S6, anti-β-TrCP and anti-β-catenin antibody were obtained from Cell Signaling Technology. Anti-luciferase antibody came from Promega, anti-nucleolin and anti-IκB antibody from Santa Cruz Biotechnology, anti-HIF-1α and anti-p21 antibody were from BD Biosciences. IRDyes 680LT and 800CM secondary antibodies were purchased from Li-COR Biosciences GmbH and horseradish peroxidase (HRP)-coupled secondary antibodies came from GE Healthcare. Blasticidin and recombinant p70^S6K1^ were from Invitrogen. Protease and phosphatase inhibitor mix were obtained from Roche. pRK7-S6K expression plasmids were kindly provided by J. Blenis. pcDNA3-β-TrCP1 was kindly provided by M. Pagano. AP-1 and NF-κB reporter plasmids were previously described [21], [22]. pGL3-Pdcd4_(39–91)_luc was previously described [23]. pRL-TK expression vector for *renilla* luciferase was from Promega.

### Cloning of Pdcd4 Constructs and Generation of Stable Cell Lines

For the generation of phosphorylation insensitive Pdcd4 constructs, serines 67, 71 and 76 were mutated to alanines in pcDNA3.1(+)-Pdcd4 plasmid [24] using the QuikChange kit (Agilent Technologies, Waldbronn, Germany) according to the manufacturer’s protocol. Reporter constructs for Pdcd4_(39–91mut)_luc were generated as previously described for Pdcd4_(39–91)_luc [23]. Briefly, a fragment of Pdcd4 (encoding amino acids 39–91) was amplified from the mutated vector. HindIII and NarI restriction sides were added to the Pdcd4-specific amplicons. The resulting fragment was fused to the luciferase expression cassette of the pGL3-control vector. The resulting Pdcd4_(mut39–91)_luc vector was used for transient transfections. For generating stable cell lines, EcoRI and BamHI restriction sides were introduced into the Pdcd4_(mut39–91)_luc pGL3-vector by PCR amplification and the resulting construct was inserted in a modified pFB-neo plasmid where the neomycin cassette has been replaced by a blasticidin resistance cassette. Stable cell lines were created by retroviral gene transfer as described before [23]. All sequences were confirmed by sequence analysis.

**Figure 2:**
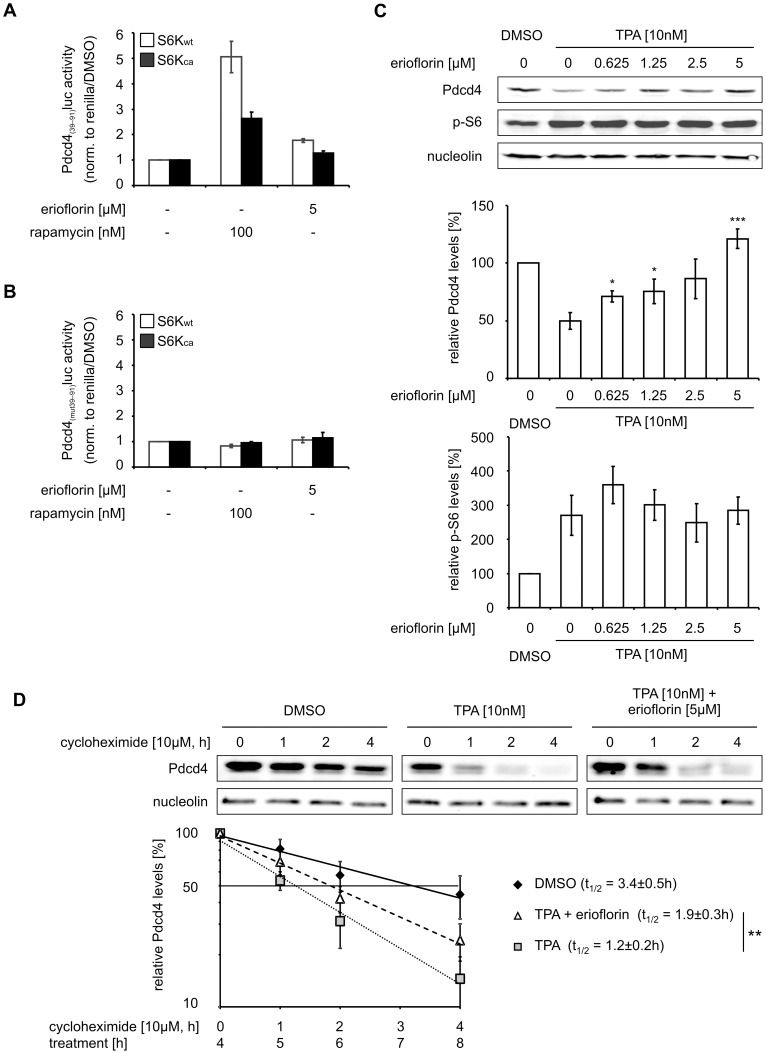
Erioflorin stabilizes Pdcd4 without affecting phosphorylation events. (A + B) HEK293 cells were transiently transfected with Pdcd4_(39–91)_luc (A) or Pdcd4_(mut39–91)_luc (B) *firefly* reporter vectors, in combination with expression vectors for either wildtype (S6K_wt_ = white bars) or constitutively active p70^S6K^ (S6K_ca_ = black bars) and a *renilla* luciferase vector one day prior to the experiment. Transfected cells were treated for 8 h with rapamycin (100 nM) or erioflorin (5 µM). *Firefly* normalized to *renilla* luciferase activity is presented relative to DMSO-treated controls. (C) HEK293 cells were treated for 8 h with TPA (10 nM) with or without erioflorin (0.625–5 µM). Whole-cell extracts were subjected to western analysis and probed with the indicated antibodies. Blots are representative of at least three independent experiments. Densitometric analysis and quantification of nucleolin-normalized Pdcd4 and phospho-S6 protein levels is shown relative to the DMSO control. (D) HEK293 cells were treated with DMSO (black diamonds), TPA (10 nM) with (white triangles) or without (gray squares) erioflorin (5 µM) for 8 h and cycloheximide (10 µM) was added for 1, 2 or 4 h. Pdcd4 protein levels were analyzed densitometrically, normalized to nucleolin and the half-life was calculated. All data are presented as means ± SEM (n≥3, *p<0.05, **p<0.01, ***p<0.001).

### Cell Culture

All cell lines (HEK, MCF7, HeLa, RKO) came from LGC Standard GmbH and were maintained in DMEM supplemented with 10% fetal bovine serum (FBS), 100 U mL^–1^ penicillin, 100 µg mL^–1^ streptomycin and 2 mM L-glutamine. HeLa cells were maintained in MEM supplemented with 10% FBS, 100 U mL^–1^ penicillin, 100 µg mL^–1^ streptomycin and 2 mM L-glutamine. Stable HEK293 Pdcd4-luc cells were maintained in regular growth medium supplemented with 3 µg mL^–1^ blasticidin. Cells were cultivated in a humidified atmosphere with 5% CO_2_ at 37°C. Medium and supplements came from PAA and FBS was purchased from Biochrom.

### Plant Material, Extraction, and Isolation

Samples of *Eriophyllum lanatum* var. *grandiflorum* (A. Gray) Jeps. were collected on a bank below a coniferous forest 27 miles east of Crescent City, CA in July 1997. The collection and identification were done by William Hess, Morton Arboretum Herbarium, Lisle, IL. A voucher specimen (collection number 0GDK760) is maintained at the Smithsonian Institution. The dried plant material (562 g) was ground and extracted by immersion in CH_2_Cl_2_-MeOH (1∶1) for 15 h in a Soxhlet apparatus [25]. The solvent was removed and the plant material was immersed for 15 h in 100% MeOH. The combined extracts were reduced to dryness *in vacuo* to give 36.8 g of crude extract. A portion of this extract (1.54 g) was subjected to a solvent-solvent partitioning scheme [26] that concentrated the Pdcd4 stabilizing activity in the ethyl acetate soluble fraction (288 mg). Size exclusion chromatography of this material on Sephadex LH-20 (2×75 cm) eluted with CH_2_Cl_2_-MeOH (1∶1) provided five major fractions (A–E). Fraction C (112 mg) was further purified by reversed-phase HPLC (Rainin; Dynamax C_18_, size 21.4×250 mm; 60 µm) eluting with a gradient of 10–100% acetonitrile in 0.05% aqueous TFA over 40 min at a flow rate of 8 mL min^–1^. Final separation by reversed-phase HPLC (Rainin; Dynamax C_18_, size 4.6×250 mm; 60 µm) eluting with a gradient of 20–40% acetonitrile in 0.05% aqueous TFA for 40 min at a flow rate of 1 mL/min provided 12.1 mg of erioflorin (0.8% yield).

### Assignment of the^ 1^H-NMR Data of Erioflorin

^1^H-NMR (600 MHz, CDCl_3_) δ 1.32 (1H, dd, *J* = 1.6, 12.6 Hz, H-9b), 1.44 (3H, s, H-14), 1.73 (1H, ddd, *J* = 2.0, 8.5, 12.4 Hz, H-2b), 1.80 (3H, d, *J* = 0.75, H-15), 1.90 (3H, s, H-4′), 2.45 (1H, dt, *J* = 3.7, 12.4 Hz, H-2a), 2.79 (1H, dd, *J = *3.7, 8.3 Hz, H-1), 2.81 (1H, dd, *J* = 3.7, 12.6 Hz, H-9a), 2.87 (1H, m, H-7), 4.48 (1H, dd, *J* = 2.0, 3.7 Hz, H-3), 5.16 (1H, m, H-8), 5.31 (1H, dq, *J* = 1.0, 9.1 Hz, H-5), 5.59 (1H, brs, H-3′), 5.75 (1H, d, *J* = 1.5 Hz, H-13a), 6.09 (1H, brs, H-3′), 6.35 (1H, d, *J* = 1.5 Hz, H-13b ), 6.65 (1H, dd, *J* = 1.6, 9.1 Hz, H-6); HREIMS *m/z* 349.1635 [M + H]^+^ (Calcd for C_19_H_25_O_6_, 349.1646).

**Figure 3:**
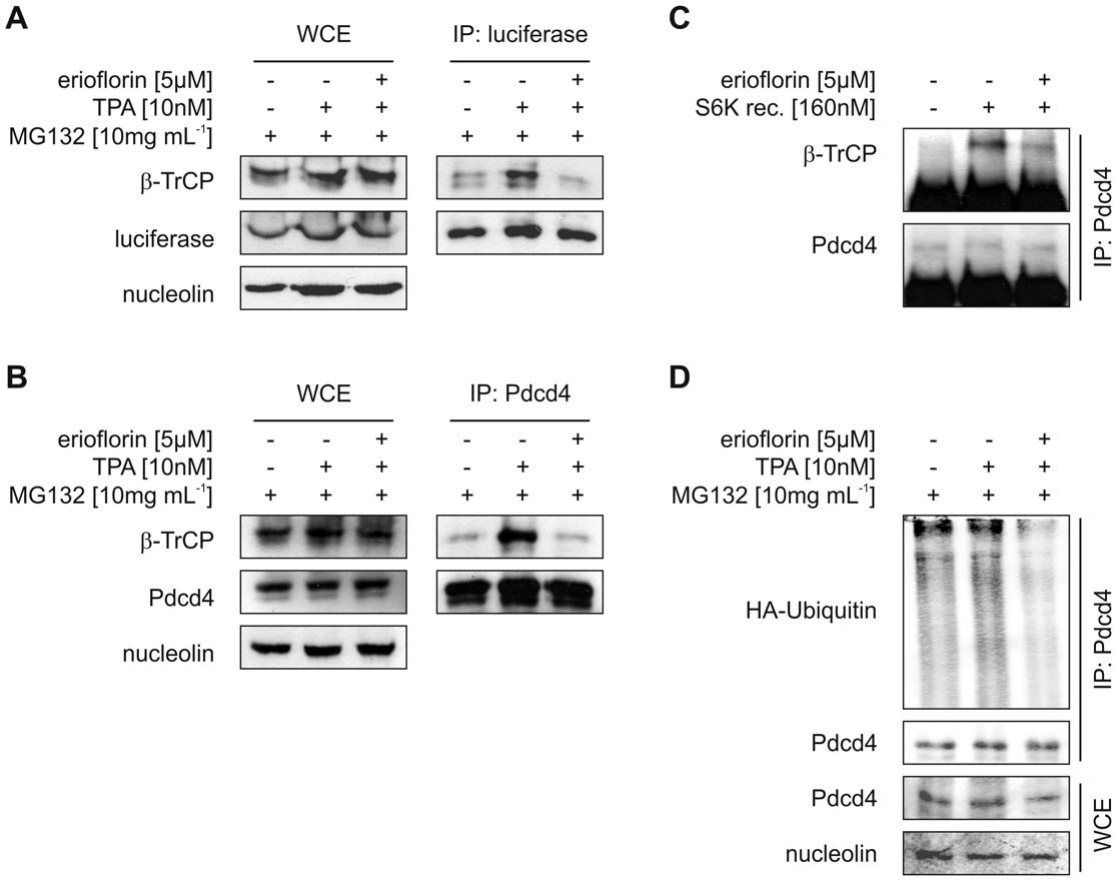
Erioflorin stabilizes Pdcd4 by interfering with β-TrCP1. (A) Stably Pdcd4_(39–91)_luc expressing or (B) wildtype HEK293 cells were transfected with a β-TrCP1 expression vector one day prior to the experiment. Transfected cells were treated for 8 h with DMSO or TPA (10 nM) with or without erioflorin (5 µM) in the presence of the proteasome inhibitor MG132 (10 mg mL^–1^). Pull-down of luciferase (A) or Pdcd4 (B) out of whole-cell extracts was performed using specific antibodies. Subsequently, immunoprecipitated (IP) proteins and whole cell extracts (WCE) were subjected to western analysis and probed with the indicated antibodies. (C) *In vitro*-transcribed/translated β-TrCP1 and Pdcd4 proteins were co-incubated for 90 min at 30°C with or without recombinant p70^S6K1^ in the absence or presence of erioflorin (5 µM). Pdcd4 protein was immunoprecipitated and β-TrCP1 binding to Pdcd4 was analyzed by western analysis. (D) HEK293 cells were transfected with a plasmid expressing HA-tagged ubiquitin. Transfected cells were treated for 8 h with DMSO or TPA (10 nM) with or without erioflorin (5 µM) in the presence of the proteasome inhibitor MG132 (10 mg mL^–1^). Proteins co-immunoprecipitated with endogenous Pdcd4 and whole-cell extracts were analyzed by western analysis with the indicated antibodies. All blots are representative for at least three independent experiments.

### Luciferase Assays

HEK293 cells stably expressing either Pdcd4_(39–91)_luc or Pdcd4_(mut39–91)_luc were seeded in a 96-well plate (1×10^4^/well) and allowed to attach for 18 h before treatment. After incubations, cells were harvested in *firefly* luciferase lysis buffer (25 mM Tris, 2 mM DTT, 1% Triton X 100, 10% glycerol, pH 7.8) and frozen at –20°C for at least 2 h. After lysis at room temperature, luminescence was measured using *firefly* luciferase substrate solution (20 mM tricine, 2.67 mM 4MgCO_3_*Mg(OH)_2_*5H_2_O, 1.07 mM MgSO_4_*7H_2_O, 100 µM EDTA, 33.3 mM dithiothreitol (DTT), 530 µM ATP, 0.213 mg mL^–1^ coenzym A, 470 mM D-luciferin) on a Mithras LB 940 (Berthold, Bad Wildbad, Germany).

p70^S6K^ expression plasmids were transiently co-transfected with pGL3-Pdcd4_(39–91)_luc or pGL3-Pdcd4_(mut39–91_)luc and a *renilla* luciferase reporter plasmid into HEK293 cells (1×10^4^) in 96-well plates using the calcium phosphate precipitation method [27]. Briefly, plasmids were incubated for 15 min at room temperature in the presence of 125 mM CaCl_2_ and HBS buffer (25 mM HEPES, 140 mM NaCl, 0.75 mM Na_2_HPO_4_, 5 mM KCl, pH 7.1) and added drop-wise to cells. Eight hours later medium was changed and incubations were continued for another 16-h period before cells were treated as indicated. Cells were lysed in passive lysis buffer (Promega, Mannheim, Germany). *Firefly* luciferase activity was determined as described above and *renilla* luciferase activity was measured with *renilla* substrate solution (0.1 M NaCl, 25 mM Tris-HCl pH 7.5, 1 mM CaCl_2_, 1 µM coelenterazine).

AP-1 and NF-κB reporter plasmids were transfected and analysis was performed as described above.

### Western Analysis

For western analysis, HEK293 cells (5×10^5^/6 cm-dish) were treated as indicated, harvested by centrifugation in ice cold PBS and lysed in lysis buffer (50 mM Tris-HCl pH 6.8, 5 mM EDTA, 6.65 M urea, 1% SDS, 10% glycerol, 1 mM phenylmethylsulfonylfluoride (PMSF), 1 x protease and phosphatase inhibitor mix, 1 mM Na_3_VO_4,_ 1 mM DTT). 80 µg protein were separated via SDS-PAGE, transferred onto nitrocellulose membranes and analyzed using specific antibodies with appropriate secondary antibodies. They were visualized using either the Odyssey infrared imaging system (Li-COR Biosciences GmbH) or enhanced chemiluminescence detection.

**Figure 4:**
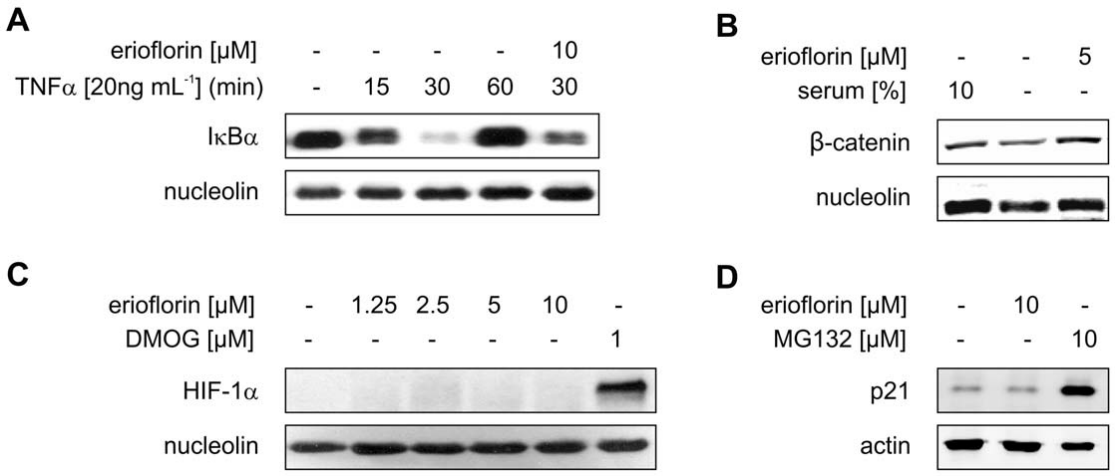
Erioflorin specifically inhibits E3-ligase β-TrCP1 activity. (A) HEK293 cells were treated with TNFα (20 ng mL^–1^) for the indicated times with or without erioflorin (10 µM). (B) HEK293 cells were maintained under full medium conditions (10% serum) or serum deprived for 24 h following treatment with erioflorin (5 µM) for 8 h. (C) HEK293 cells were treated for 8 h with erioflorin (1.25–10 µM) or the prolyl-hydroxylase inhibitor dimethyloxallylglycine (DMOG, 1 µM). (D) HeLa cells were serum deprived for 48 h prior to treatment with erioflorin (10 µM) or the proteasome inhibitor MG132 (10 µM) for 8 h. Whole cell extracts were subjected to western analysis and probed with the indicated antibodies. Blots are representative for at least three independent experiments.

### Immunoprecipitation Assays

For immunoprecipitation assays, HEK293 cells (1×10^6^/10 cm-dish) were transiently transfected with β-TrCP1 expression plasmid or with a plasmid expressing HA-tagged ubiquitin as described above. One day after transfection, cells were treated as indicated. After treatment, cells were lysed on ice for 30 min in immunoprecipitation (IP) buffer (50 mM Tris-HCl pH 7.4, 300 mM NaCl, 5 mM EDTA, 1% NP-40, 1 mM PMSF, 1 x protease and phosphatase inhibitor mix, 1 mM Na_3_VO_4_). 1 mg protein was incubated with either 5 µL anti-Pdcd4- or 5 µL anti-luciferase-antibody in 300 µL IP buffer for 6 h. Then, 20 µL 50% slurry of Protein A Sepharose (Sigma-Aldrich) were added and incubated overnight. Sepharose was precipitated by centrifugation and washed three times with IP buffer. Proteins were eluted by addition of 2 x loading buffer and incubation at 95°C for 5 min. Immunoprecipitated proteins were separated via SDS-PAGE and visualized using western analysis with the indicated antibodies. Whole cell extracts served as loading control and analysis was performed as described above.

### *In Vitro*-Transcription/Translation Assay

Pdcd4 and β-TrCP1 proteins were generated by *in vitro*-transcription/translation from pcDNA3.1(+)-Pdcd4 and pcDNA3-β-TrCP1, respectively, using the TNT Coupled Reticulocyte Lysate System from Promega according to manufacturer’s protocol. Interaction reactions were performed for 90 min at 30°C in a volume of 15 µL containing 50 mM Tris-HCl pH 7.6, 5 mM MgCl_2_, 2 mM ATP, 4 µL Pdcd4, 3 µL β-TrCP1 and 160 nM recombinant p70^S6K1^ as indicated. Immunoprecipitation and western analyses were performed as described above.

**Figure 5:**
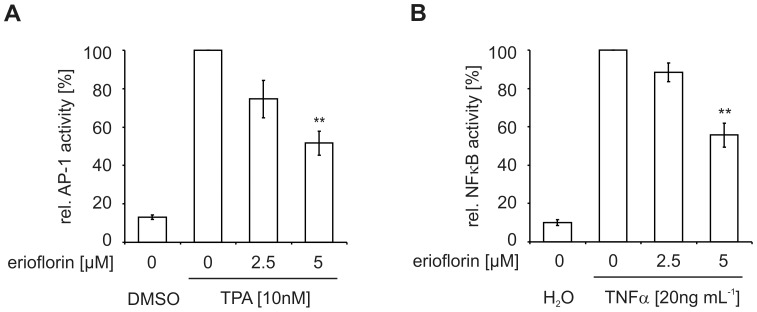
Erioflorin inhibits AP-1- and NF-κB-trancriptional activities. (A) HEK293 cells were co-transfected with an AP-1 *firefly* reporter and a *renilla* luciferase plasmid one day prior to experiments. Transfected cells were treated for 16 h with TPA (10 nM) with or without erioflorin (2.5 and 5 µM). Relative AP-1 activity was normalized to *renilla* luciferase and presented relative to TPA-only treated cells. (B) HEK293 cells were co-transfected with a NF-κB *firefly* reporter and a *renilla* luciferase plasmid one day prior to experiments. Transfected cells were treated for 16 h with TNFα (20 mg mL^–1^) with or without erioflorin (2.5 and 5 µM). Relative NF-κB activity was normalized to *renilla* luciferase and presented relative to TNFα-only treated cells. All data are given as means ± SEM (n≥3, **p<0.01).

### Viability Assay

HEK293 cells (1×10^4^/well) were seeded in a 96-well plate and allowed to attach for 18 h. Cells were treated as indicated and viability was measured using the CellTiter-Glo Luminescent Cell Viability Assay from Promega according to the manufacturer’s protocol.

### Proliferation Assay

RKO, HeLa and MCF7 cells (1×10^4^/well) were seeded in 96-well plates one day prior to the experiment. Cells were treated as indicated and analyzed in an IncuCyte® Live-Cell Imaging System (Essen Bioscience) by microscopic determination of monolayer confluency.

**Figure 6:**
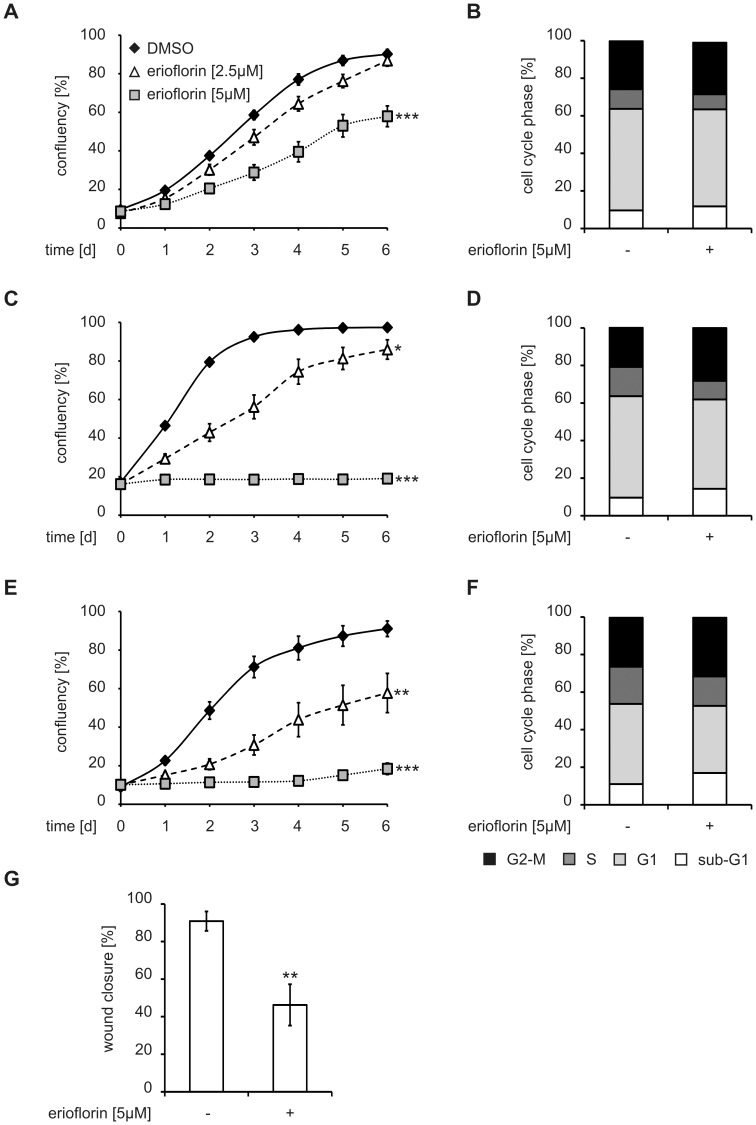
Erioflorin inhibits cell proliferation and alters cell cycle progression. (A, C, E) MCF7, HeLa and RKO cells were seeded at 10–20% confluency one day prior to the experiment and treated with DMSO (black diamonds) or erioflorin (2.5 (white triangles) and 5 µM (gray squares)). Cell confluency was followed for six days. (B, D, F) MCF7, HeLa and RKO cells were serum deprived for 48 h and treated with erioflorin (5 µM) for 16 h. After propidium iodide staining, distribution of the cells to the different phases of the cell cycle (subG1 (white), G1 (light gray), S (dark gray), G2/M (black)) was determined. (G) RKO colon carcinoma cells were subjected to a scratch wound assay. After administration of the scratch, medium was changed to control or erioflorin (5 µM) containing medium. Wound closure was measured after 24 h and relative wound closure is given as the ratio of the width of the scratch at 24 h and to 0 h. All data are given as means ± SEM (n≥3, *p<0.05, **p<0.01, ***p<0.001).

### Cell Cycle Analysis

RKO, HeLa and MCF7 cells (1×10^5^/3 cm-dish) were treated as indicated. Cells were harvested by centrifugation in PBS, lysed in PBS^+^ (PBS including 1.1 g L^-1^ glucose and 0.5 mM EDTA) and centrifuged again. Pellets were incubated in PBS^+^ + RNase (PBS^+^ including 50 µg mL^–1^ RNase) for 15 min. DNA was stained by incubation for 15 min with 10% NP-40 and 10 µg propidium iodide. Stained cells were analyzed using a LSR Fortessa flow cytometer (BD Bioscience). Cell cycle phase distribution was calculated with FlowJo software.

### Scratch Wound Assay

RKO cells (5×10^5^/well) were seeded in 24-well plates. Upon reaching 100% confluency scratches were administered using a 10 µL pipet tip. After removal of medium and floating cells, cells were treated as indicated and pictures were taken using a Zeiss microscope Axiovert 200 M. Wound closure was calculated as scratch width at 24 h relative to initial wound size.

### Statistical Analysis

Each experiment was performed at least three times. Representative blots are shown. Data are presented as means ± SEM. Significance analysis was performed using Student’s t-test.

## Results and Discussion

### Erioflorin Stabilizes Pdcd4 from TPA-Induced Degradation

We have previously shown that TPA induces the phosphorylation-dependent proteasomal degradation of Pdcd4 [5] and further introduced a luciferase-based assay to identify compounds stabilizing Pdcd4 from TPA-induced degradation [23], [28], [29]. Briefly, a vector spanning the domain containing both the p70^S6K1^-phosphorylation and the β-TrCP-recognition motifs (aa 39–91) fused to luciferase (Pdcd4_(39–91)_luc) served as a sensitive tool to analyze TPA-induced Pdcd4 degradation. As natural products provide a rich source for the development of novel therapeutics, which is supported by the fact that 50% of all small molecule drugs introduced to the market between 1981 and 2008 (>60% for cancer therapeutics) were either natural products or derived from natural products [30], we carried out a high-throughput screen of 135,678 natural product extracts using this approach. We identified an extract from the woolly sunflower *Eriophyllum lanatum* (Asteraceae) to increase the luciferase signal relative to TPA-treated controls. Sequential fractionation of the extract by solvent partitioning, size-exclusion chromatography, and C_18_ HPLC provided erioflorin as the active agent. Erioflorin is a sesquiterpene lactone possessing a tricyclic germacranolide skeleton (Figure 1A). The structure was established by spectroscopic analyses and comparison of its spectral data with values reported in the literature. A complete assignment of the ^1^H NMR data for erioflorin was made as only a partial assignment has been published to date [31]. Our data corresponded closely to those reported for heliangine [32], a structurally related germacranolide sesquiterpene that only differs from erioflorin by the composition of the ester side-chain at C-8. The ^13^C NMR spectral data we recorded for erioflorin were fully consistent with previously reported values [33]. The relative configuration of erioflorin was confirmed by extensive ROESY correlation data.

Dose-response studies revealed that erioflorin significantly rescued Pdcd4 from 8 h TPA-induced degradation at concentrations as low as 1.25 µM (36.7±7.5%) (Figure 1B). For a detailed calculation of the Pdcd4_(39–91)_luc stabilizing activity see Figure S1A and B. Maximal recovery of luciferase activity was achieved with 5 µM erioflorin (75.5±7.4%). At concentrations at or above 10 µM erioflorin luciferase activity was markedly reduced. HEK293 cells stably expressing the Pdcd4_(39–91)_luc construct harboring S67/71/76A mutations (Pdcd4_(mut39–91)_luc), i.e. an inactive phospho-degron, which prevents phosphorylation and degradation, were used to determine nonspecific effects. As no significant reduction of the luciferase signal in response to TPA-only treatment in these cells was detected, activity was calculated relative to TPA-only treated cells (Figure S1C). As shown in Figure 1C, erioflorin concentrations up to 5 µM, when co-incubated with TPA, did not affect luciferase activity, whereas concentrations at or above 10 µM significantly decreased the luciferase signal. As viability of the cells was not affected at these concentrations of erioflorin in combination with TPA (Figure S3A), the loss of luciferase signal was attributed to nonspecific inhibition of the luciferase vectors. This observation is in accordance with a previous report showing that other sesquiterpene lactones, such as parthenolide, inhibit *firefly* luciferase activity [34].

Based on these results, we propose that erioflorin potently stabilizes Pdcd4_(39–91)_luc, as an indicator of Pdcd4 protein stability, from TPA-induced degradation.

### Erioflorin Stabilizes Pdcd4 not via p70^S6K1^ Inactivation

In line with the concept that TPA-induced degradation of Pdcd4 requires active PI3K-mTOR-p70^S6K^ signaling [5], [11], inhibition of p70^S6K^ [35] or upstream factors such as BCR/ABL [36] was previously shown to block Pdcd4 degradation. Thus, we next analyzed the effect of erioflorin on p70^S6K^ activity. To this end, HEK293 cells were transiently co-transfected with expression vectors for wild type p70^S6K^ (S6K_wt_) or a constitutively active p70^S6K^ mutant (S6K_ca_) in combination with either the degradable Pdcd4_(39–91)_luc or the stable Pdcd4_(mut39–91)_luc construct. The activity of the Pdcd4_(mut39–91)_luc construct was generally higher than the activity of the Pdcd4_(39–91)_luc construct when either S6K_wt_ or S6K_ca_ were overexpressed (Figure S2), which verifies that the intact phospho-degron is required to respond to p70^S6K^. Since p70^S6K1^ requires activation by the upstream kinase mTOR, the mTOR inhibitor rapamycin was expected to affect the activity of S6K_wt_ but not of S6K_ca_. As anticipated, rapamycin (100 nM) increased Pdcd4_(39–91)_luc activity significantly more in S6K_wt_ than in S6K_ca_ expressing cells. In contrast, erioflorin showed no significant difference in its ability to stabilize the Pdcd4_(39–91)_luc signal under S6K_wt_ or S6K_ca_ co-expressing conditions (Figure 2A), which indicates that erioflorin enhances Pdcd4 stability downstream of p70^S6K^ activity. Pdcd4_(mut39–91)_luc activity was neither affected by S6K_wt_ nor by S6K_ca_ and consequently remained unaffected by treatment with rapamycin or erioflorin (Figure 2B).

These observations serve as a first indication that erioflorin stabilizes Pdcd4 without interfering with p70^S6K^ activity that is required for its phosphorylation.

### Erioflorin Protects Endogenous Pdcd4 from TPA-Induced Degradation

To confirm that erioflorin not only stabilizes Pdcd4_(39–91)_luc from TPA-induced degradation but is also effective at the level of endogenous Pdcd4 protein, we next analyzed Pdcd4 protein expression upon TPA treatment with or without erioflorin. Figure 2C (upper panel) shows that Pdcd4 protein was markedly reduced when HEK293 cells were exposed to TPA (10 nM) for 8 h. Erioflorin rescued Pdcd4 from TPA-induced degradation at low micromolar concentrations. Quantitative analysis revealed that Pdcd4 protein was reduced to 49.9±7.1% of the DMSO control in response to TPA treatment. Erioflorin rescued Pdcd4 protein expression from TPA-induced loss in a concentration-dependent manner from 71.1±4.7% at 0.625 µM to 121.1±8.6% at 5 µM as compared to the DMSO control (Figure 2C, middle panel). Importantly, erioflorin rescued Pdcd4 protein levels from TPA-induced degradation in the tumor cell lines MCF7 (breast) and RKO (colon) in a similar fashion (Figure S4). Thus, erioflorin-mediated Pdcd4 stabilization appears to be a general mechanism rather than a cell line specific phenomenon. Furthermore, blocking *de novo* protein synthesis with cycloheximide (10 µM), revealed that TPA-restricted Pdcd4 protein half-life (1.2±0.2 h) was significantly extended by erioflorin co-treatment (1.9±0.3 h) (Figure 2D). To verify that this rescue was indeed independent of an effect on p70^S6K1^ activity, we analyzed phosphorylation of a prototypical p70^S6K1^-target, ribosomal protein S6. While S6-phosphorylation was increased in response to TPA to 269.9±58.4% of the DMSO control, erioflorin did not significantly change TPA-induced S6-phosphorylation (Figure 2C, upper and lower panel).

Taken together, we conclude that erioflorin stabilizes endogenous Pdcd4 from TPA-induced degradation not by interfering with the phosphorylation of the latter, but rather by mechanisms affecting the degradation of already phosphorylated Pdcd4.

### Erioflorin Disrupts the β-TrCP1/Pdcd4-Interaction

As phosphorylated Pdcd4 is recognized by the E3-ubiquitin ligase β-TrCP1, which mediates polyubiquitination and subsequent proteasomal degradation of its targets [11], we next determined the effect of erioflorin on the intracellular binding of β-TrCP1 to Pdcd4. Binding of transiently overexpressed β-TrCP1 to Pdcd4_(39–91)_luc increased upon treatment with TPA (8 h, 10 nM). Erioflorin (5 µM) significantly diminished the TPA-induced interaction between Pdcd4_(39–91)_luc and β-TrCP1 (Figure 3A, right panel). Similarly, β-TrCP1 co-immunoprecipitated with endogenous Pdcd4 in response to TPA (8 h, 10 nM), which again was significantly attenuated by 5 µM erioflorin (Figure 3B, right panel). To verify that erioflorin directly targets the interaction of Pdcd4 with β-TrCP1, we studied the effect of erioflorin on β-TrCP1-Pdcd4-binding *in vitro*. As anticipated, *in vitro*-transcribed/translated β-TrCP1 bound to *in vitro*-transcribed/translated Pdcd4 only in the presence of recombinant p70^S6K^. This binding was markedly reduced in the presence of erioflorin (Figure 3C). This observation not only supports inhibition of the interaction between β-TrCP1 and its target Pdcd4 by erioflorin, but also suggests that inhibition of additional factors within the proteasomal degradation machinery is not required for erioflorin-dependent stabilization of Pdcd4. As E3-ligases mediate ubiquitination of interacting target-proteins, we next addressed if erioflorin affects TPA-induced ubiquitination of Pdcd4. Therefore, binding of transiently expressed HA-ubiquitin to Pdcd4 was determined by immunoprecipitation. Indeed, Pdcd4 ubiquitination in TPA-treated cells (10 nM, 8 h) was reduced dramatically by co-treatment with erioflorin (5 µM) (Figure 3D).

Taken together, these data indicate that erioflorin stabilizes Pdcd4 by interfering with the interaction of Pdcd4 with the E3-ligase β-TrCP1, without affecting Pdcd4 phosphorylation.

### Erioflorin Specifically Stabilizes Targets of the E3-Ligase β-TrCP

In an attempt to gain insights into the selectivity of erioflorin, we questioned whether erioflorin only interferes with the interaction between Pdcd4 and β-TrCP or if other β-TrCP targets are stabilized as well. Among the numerous proteins targeted for degradation by β-TrCP [37], IκBα is among the best characterized ones [38]. When IκBα is phosphorylated by IKKs it is recognized and bound by β-TRCP and marked for proteasomal degradation, allowing for activation of the transcription factor NF-κB [39]. In line, activation of IKKs by TNFα (20 ng mL^–1^) led to the rapid, but transient decrease in IκBα protein levels in HEK293 cells (Figure 4A). Reduced IκBα was observed already after 15 min and was maximal at 30 min of TNFα treatment (lane 3). Longer treatments with TNFα (60 min) allowed for a complete recovery of the protein. Erioflorin (10 µM) stabilized IκBα at 30 min of TNFα (compare lanes 1, 3 and 5). Similarly, erioflorin (8 h, 5 µM) stabilized the β-TrCP-target β-catenin, which is phosphorylated by the glycogen synthase kinase 3β (GSK3β) [40], from serum deprivation-induced degradation (Figure 4B). These results suggest that erioflorin does not interfere exclusively with the interaction of Pdcd4 and β-TrCP, instead it appears to stabilize various β-TrCP-targets. We next determined if targets of other E3-ligases are affected by erioflorin as well. Therefore, we analyzed the effect of erioflorin on the stability of HIF-1α protein, which is targeted for proteasomal degradation by the E3-ligase von Hippel-Lindau protein (pVHL) [41]. Inhibition of the prolyl-hydroxylases, which mark HIF-1α for interaction with pVHL, using 1 µM dimethyloxalylglycine (DMOG) for 8 h resulted in strong accumulation of HIF-1α protein (Figure 4C). However, erioflorin (up to 10 µM) did not stabilize HIF-1α. Along the same line, p21, a target of the closely related SCF-E3-ligase Skp2 [42], strongly accumulated when proteasomal degradation was blocked with MG132 (10 µM) for 8 h whereas it did not increase in response to erioflorin (5 µM) (Figure 4D). Thus, we propose that erioflorin stabilizes β-TrCP targets by interfering with the interaction of β-TrCP1 with them, while leaving targets of other E3-ligases unaffected. The fact that erioflorin did not stabilize targets of other E3-ligases can be taken as an indicator that it does not affect enzymes that would be expected to affect broader target spectra such as E1- or E2-enzymes. Importantly, further studies are required to establish the exact site of action of erioflorin, i.e. if it directly interacts with β-TrCP or rather with phospho-degrons on the target-proteins.

Taken together, our results imply that erioflorin specifically inhibits the interaction of the E3-ligase β-TrCP1 with various of its targets, while it does not affect the stability of proteins regulated by other E3-ligases. E3-ubiquitin ligases have been increasingly appreciated as powerful new therapeutic targets in recent years [43], [44]. MLN4924 was identified to inhibit cullin-RING E3-ubiquitin ligases (CRL) by attenuating the NEDD8-activating enzyme and thus, preventing neddylation of CRLs [45]. In addition, various compounds have been identified to inhibit the activity of or the interaction with targets of specific E3-ligases such as Skp2 [46], Mdm2 [47], Met30 [48] or Cdc4 [49]. Yet, erioflorin appears as the first compound interfering with the degradation of various β-TrCP1 targets such as Pdcd4.

### Erioflorin Reduces AP-1- and NF-κB-Dependent Transcription

As β-TrCP was suggested to be pro-tumorigenic, despite its broad target-protein spectrum including both pro- (β-catenin) and anti-oncogenic proteins (Pdcd4, IκBs, BimEL) [37], we next aimed at determining the functional, tumor-related consequences of erioflorin. Initially, we analyzed its impact on the activity of the tumor-associated transcription factors AP-1 and NF-κB. AP-1 was chosen based on previous reports, which showed that Pdcd4 affects AP-1 activity [24], [50], while NF-κB was selected since it is the direct target of the above analyzed IκBα [39]. To this end, HEK293 cells were transiently transfected with an AP-1 reporter vector one day prior to the experiment. Treatment with TPA (10 nM, 16 h) strongly induced AP-1 activity. Erioflorin reduced TPA-induced AP-1 activity to 74.6±9.7% at 2.5 µM and to 51.7±6.2% at 5 µM (Figure 5A). This effect was not due to toxicity as TPA in combination with erioflorin (up to 20 µM) was not toxic in this setting (Figure S3A). To assess the impact of erioflorin on NF-κB activity, HEK293 cells were transiently transfected with a NF-κB reporter vector one day prior to the experiment. Treatment with TNFα (20 ng mL^–1^, 16 h) strongly induced NF-κB activity. While erioflorin co-treatment only slightly inhibited TNFα-induced NF-κB activity at 2.5 µM, 5 µM erioflorin sufficed to significantly reduce the activity to 55.7±6.3% of TNFα-only treated cells (Figure 5B). Again, erioflorin (up to 5 µM) in combination with TNFα did not adversely affect cell viability in these cells (Figure S3B).

These results imply that erioflorin not only stabilizes β-TrCP-target-proteins, but also inhibits transcriptional activity of downstream effectors such as AP-1 and NF-κB.

### Erioflorin Shows Anti-Proliferative Potential *In Vitro*

Both AP-1 and NF-κB have been extensively shown to play an important role in tumorigenesis and tumor cell proliferation. Therefore, we next addressed the influence of erioflorin on the proliferation of various cancer cell lines. Erioflorin treatment (2.5 and 5 µM) reduced proliferation of MCF7 (weakly), HeLa (moderately-strongly) and RKO cells (strongly) (Figure 6A, C, E). This is in line with a recent report demonstrating that inhibition of β-TrCP by genetic means attenuated viability and proliferation of breast cancer cells and rendered them more sensitive to various anticancer drugs [18]. Interestingly, cell cycle analysis revealed that, while MCF7 cells showed only minor changes in cell cycle distribution in response to erioflorin (5 µM), both HeLa and RKO cells displayed a pronounced increase in G2/M- and subG1-phases, whereas G1- and S-phases were reduced (Figure 6B, D, F and Table S1). These findings closely correlate with our observations on proliferation, where MCF7 cells also appeared to be least sensitive. Interestingly, while the increase in sub-G1, i.e. apoptosis, in the tumor cell lines (RKO>HeLa>MCF7) in response to erioflorin appears to contradict previous observations that the viability of HEK293 cells was not affected by erioflorin (Figure S3), this might be due to different experimental conditions and/or cell types. Specifically, the lack of toxicity in HEK293 cells fits well with the observed sequence of sensitivities, i.e. more advanced tumor cells (e.g. RKO) being more sensitive to erioflorin than non-invasive tumor cells (e.g. MCF7) or non-tumorigenic cells (e.g. HEK293). Therefore, we lastly performed scratch wound assays using the highly invasive, yet most erioflorin-sensitive colon carcinoma cell line RKO. RKO cells efficiently closed (90.9±5.2%) scratches in confluent cell layers at 24 h, whereas erioflorin (5 µM) significantly inhibited wound closure to 53.8±11.0% (Figure 6G). These observations further strengthen the notion that disrupting the interaction between β-TrCP and its targets could open a novel avenue for the development of future anti-cancer therapeutics.

In summary, these data show that erioflorin suppresses tumor-associated events such as activation of AP-1- and NF-κB-dependent transcription and in addition alters cell cycle progression resulting in inhibition of proliferation in tumor cells. Interestingly, erioflorin appears to affect proliferation and cell cycle progression preferentially in more advanced tumor cell lines as compared to non-invasive lines or non-tumorigenic lines, which might be interest with respect to the development of future tumor therapeutics targeting β-TrCP.

### Conclusion

This is the first report characterizing a natural product, small molecule compound that tumor suppressor Pdcd4 by interrupting its interaction with the E3-ligase β-TrCP. As erioflorin selectively stabilizes a number of β-TrCP targets leaving targets of other E3-ligases unaffected, it appears to functionally work as a β-TrCP inhibitor. We further provide evidence that erioflorin holds anti-tumorigenic potential as it inhibits AP-1 and NF-κB transcriptional activity, and interferes with cell cycle progression and proliferation of tumor cells. Based on the distinct set of proteins targeted by β-TrCP1 for degradation, we predict that erioflorin will generate less adverse side effects as compared to general proteasome inhibitors currently used in tumor therapy.

## Supporting Information

Figure S1 **Conversion of relative Pdcd4_(39–91)_luciferase light units to relative Pdcd4_(39–91)_ stabilizing activity.** (A) Stably Pdcd4_(39–91)_luc expressing HEK293 cells were treated for 8 h with TPA (10 nM) with or without rapamycin (100 nM). Relative light units (RLU) of the Pdcd4_(39–91)_luciferase fusion protein were normalized to DMSO. (B) Experiment from (A). Pdcd4_(39–91)_ stabilizing activity of rapamycin was determined relative to Δ(RLU_control_–RLU_TPA-only_). (C) Stably Pdcd4_(mut39–91)_luc expressing HEK293 cells were treated as in (A). Luciferase activity is given relative to TPA-treated controls.
(DOC)

Figure S2 **Expression of Pdcd4_(39–91)_luc and Pdcd4_(mut39–91)_luc in the presence of overexpressed p70^S6K^.** HEK293 cells were transiently transfected with Pdcd4_(39**–**91)_luc (left bars) or Pdcd4_(mut39**–**91)_luc (right bars) *firefly* reporter vectors, in combination with expression vectors for either wildtype (S6K_wt_ = white bars) or constitutively active p70^S6K^ (S6K_ca_ = black bars) and a *renilla* luciferase vector one day prior to the experiment. *Firefly* luciferase levels were normalized to *renilla* luciferase and presented relative to Pdcd4_(39**–**91)_luc/S6K_wt_ (relative light units = RLU).
(DOC)

Figure S3 **Erioflorin does not influence cell viability in combination with TPA or TNFα.** (A) HEK293 cells were treated for 16 h with TPA (10 nM) with or without erioflorin (2.5 to 20 µM). Cell viability was analyzed using the CellTiter glow assay (Promega) according to manufacturer’s protocol and is given relative to DMSO-treated controls. (B) HEK293 cells were treated for 16 h with TNFα (20 ng mL^–1^) or with or without erioflorin (2.5 and 5 µM). Cell viability was analyzed as described in A.
(DOC)

Figure S4 **Erioflorin stabilizes Pdcd4 in breast and colon carcinoma cells.** (A) MCF7 and (B) RKO cells were treated for 8 h with TPA (10 nM) with or without erioflorin (10 µM). Whole-cell extracts were subjected to Western analysis and probed with the indicated antibodies.
(DOCX)

Table S1 Cell cycle distribution in response to erioflorin.
(DOC)
