# Supplementary material for: Erioflorin Stabilizes the Tumor Suppressor Pdcd4 by Inhibiting Its Interaction with the E3-ligase β-TrCP1
Source: PLoS One. 2012 Oct 2;7(10):e46567. doi: 10.1371/journal.pone.0046567 (PMC3462793; doi:10.1371/journal.pone.0046567)
Supplement: Figure S1 — Conversion of relative Pdcd4(39–91)luciferase light units to relative Pdcd4(39–91) stabilizing activity. (A) Stably Pdcd4(39–91)luc expressing HEK293 cells were treated for 8 h with TPA (10 nM) with or without rapamycin (100 nM). Relative light units (RLU) of the Pdcd4(39–91)luciferase fusion protein were normalized to DMSO. (B) Experiment from (A). Pdcd4(39–91) stabilizing activity of rapamycin was determined relative to Δ(RLUcontrol–RLUTPA-only). (C) Stably Pdcd4(mut39–91)luc expressing HEK293 cells were treated as in (A). Luciferase activity is given relative to TPA-treated controls. (DOC) [file pone.0046567.s001.doc]

**
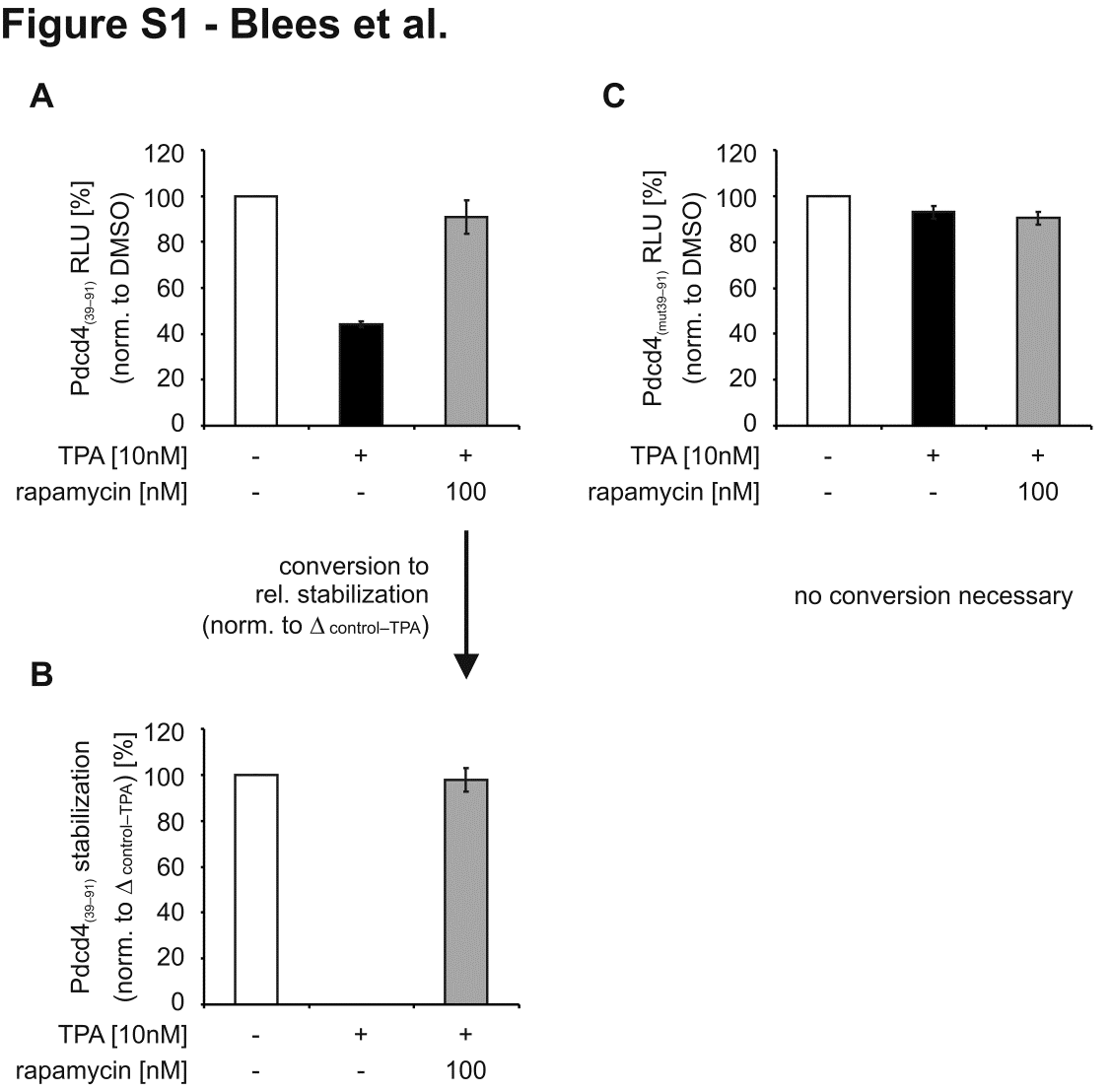
**

**Figure S1. Conversion of relative Pdcd4_(39–91)_luciferase light units to relative Pdcd4_(39–91)_ stabilizing activity.** (A) Stably Pdcd4_(39–91)_luc expressing HEK293 cells were treated for 8h with TPA (10 nM) with or without rapamycin (100 nM). Relative light units (RLU) of the Pdcd4_(39–91)_luciferase fusion protein were normalized to DMSO. (B) Experiment from (A). Pdcd4_(39–91)_ stabilizing activity of rapamycin was determined relative to Δ(RLU_control_–RLU_TPA-only_). (C) Stably Pdcd4_(mut39–91)_luc expressing HEK293 cells were treated as in (A). Luciferase activity is given relative to TPA-treated controls.
