# Supplementary material for: Erioflorin Stabilizes the Tumor Suppressor Pdcd4 by Inhibiting Its Interaction with the E3-ligase β-TrCP1
Source: PLoS One. 2012 Oct 2;7(10):e46567. doi: 10.1371/journal.pone.0046567 (PMC3462793; doi:10.1371/journal.pone.0046567)
Supplement: Figure S2 — Expression of Pdcd4(39–91)luc and Pdcd4(mut39–91)luc in the presence of overexpressed p70S6K. HEK293 cells were transiently transfected with Pdcd4(39–91)luc (left bars) or Pdcd4(mut39–91)luc (right bars) firefly reporter vectors, in combination with expression vectors for either wildtype (S6Kwt = white bars) or constitutively active p70S6K (S6Kca = black bars) and a renilla luciferase vector one day prior to the experiment. Firefly luciferase levels were normalized to renilla luciferase and presented relative to Pdcd4(39–91)luc/S6Kwt (relative light units = RLU). (DOC) [file pone.0046567.s002.doc]

**
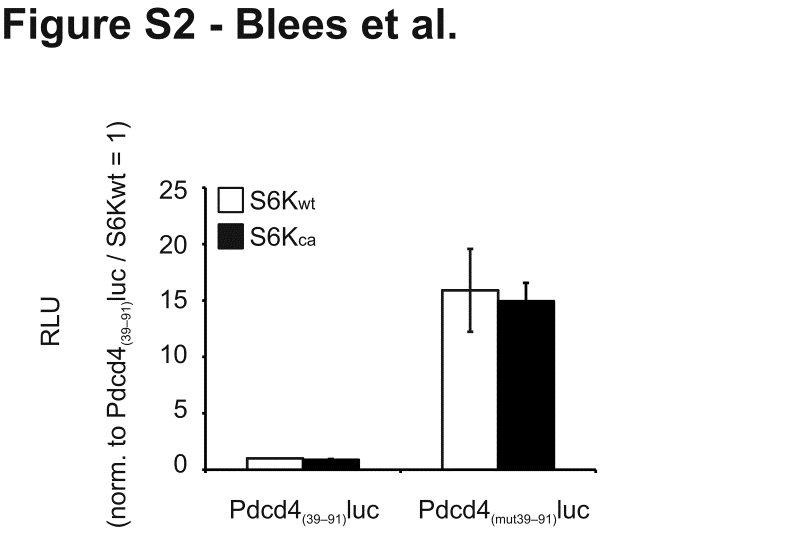
**

**Figure S2.** **Expression of Pdcd4_(39–91)_luc and Pdcd4_(mut39–91)_luc in the presence of overexpressed p70^S6K^.** HEK293 cells were transiently transfected with Pdcd4_(39_**–**_91)_luc (left bars) or Pdcd4_(mut39_**–**_91)_luc (right bars) *firefly* reporter vectors, in combination with expression vectors for either wildtype (S6K_wt_ = white bars) or constitutively active p70^S6K^ (S6K_ca_ = black bars) and a *renilla* luciferase vector one day prior to the experiment. *Firefly* luciferase levels were normalized to *renilla* luciferase and presented relative to Pdcd4_(39_**–**_91)_luc / S6K_wt_ (relative light units = RLU).
