# Supplementary material for: Erioflorin Stabilizes the Tumor Suppressor Pdcd4 by Inhibiting Its Interaction with the E3-ligase β-TrCP1
Source: PLoS One. 2012 Oct 2;7(10):e46567. doi: 10.1371/journal.pone.0046567 (PMC3462793; doi:10.1371/journal.pone.0046567)
Supplement: Figure S3 — Erioflorin does not influence cell viability in combination with TPA or TNFα. (A) HEK293 cells were treated for 16 h with TPA (10 nM) with or without erioflorin (2.5 to 20 µM). Cell viability was analyzed using the CellTiter glow assay (Promega) according to manufacturer’s protocol and is given relative to DMSO-treated controls. (B) HEK293 cells were treated for 16 h with TNFα (20 ng mL–1) or with or without erioflorin (2.5 and 5 µM). Cell viability was analyzed as described in A. (DOC) [file pone.0046567.s003.doc]

**
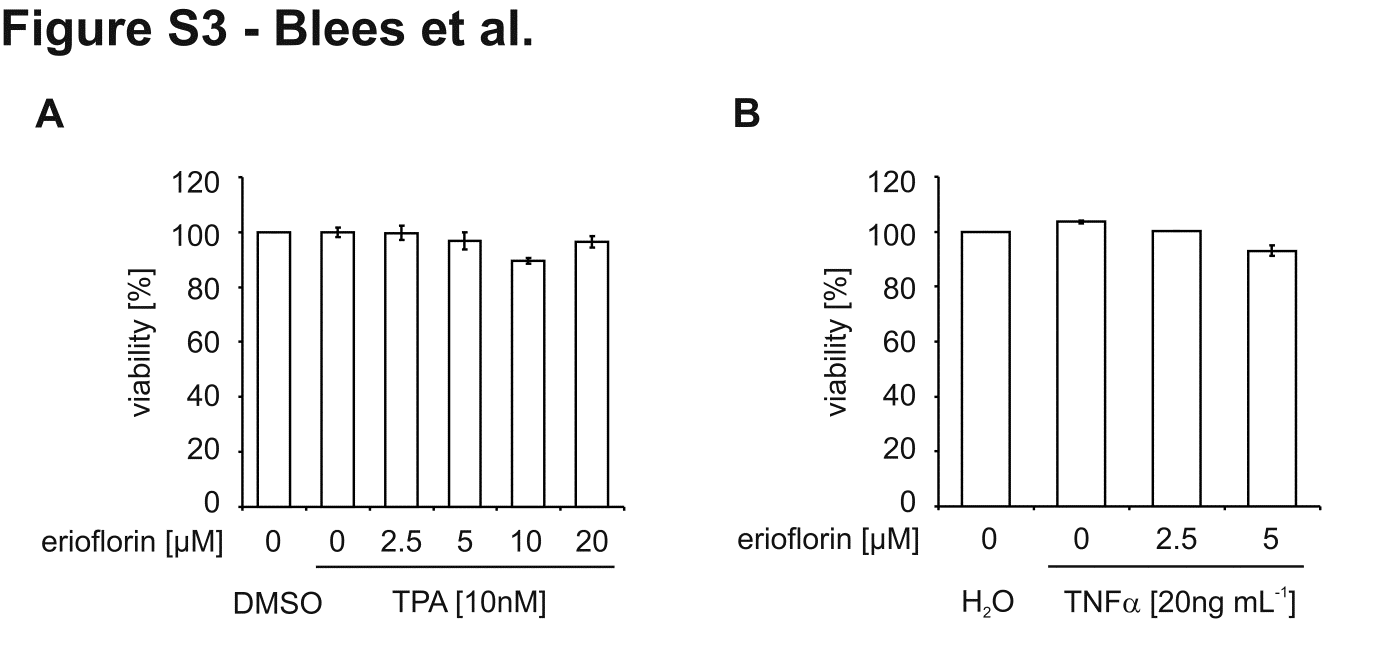
Figure S3. Erioflorin does not influence cell viability in combination with TPA or TNFα.** (A) HEK293 cells were treated for 16h with TPA (10 nM) with or without erioflorin (2.5 to 20 µM). Cell viability was analyzed using the CellTiter glow assay (Promega) according to manufacturer’s protocol and is given relative to DMSO-treated controls. (B) HEK293 cells were treated for 16h with TNFα (20 ng mL^–1^) or with or without erioflorin (2.5 and 5 µM). Cell viability was analyzed as described in A.
