# Supplementary material for: Erioflorin Stabilizes the Tumor Suppressor Pdcd4 by Inhibiting Its Interaction with the E3-ligase β-TrCP1
Source: PLoS One. 2012 Oct 2;7(10):e46567. doi: 10.1371/journal.pone.0046567 (PMC3462793; doi:10.1371/journal.pone.0046567)
Supplement: Figure S4 — Erioflorin stabilizes Pdcd4 in breast and colon carcinoma cells. (A) MCF7 and (B) RKO cells were treated for 8 h with TPA (10 nM) with or without erioflorin (10 µM). Whole-cell extracts were subjected to Western analysis and probed with the indicated antibodies. (DOCX) [file pone.0046567.s004.docx]

**

**

**Figure S4. Erioflorin stabilizes Pdcd4 in breast and colon carcinoma cells.** (A) MCF7 and (B) RKO cells were treated for 8h with TPA (10 nM) with or without erioflorin (10 μM). Whole-cell extracts were subjected to Western analysis and probed with the indicated antibodies.
