# Supplementary figures and images for: Erioflorin Stabilizes the Tumor Suppressor Pdcd4 by Inhibiting Its Interaction with the E3-ligase β-TrCP1
Source: PLoS One. 2012 Oct 2;7(10):e46567. doi: 10.1371/journal.pone.0046567 (PMC3462793; doi:10.1371/journal.pone.0046567)

**
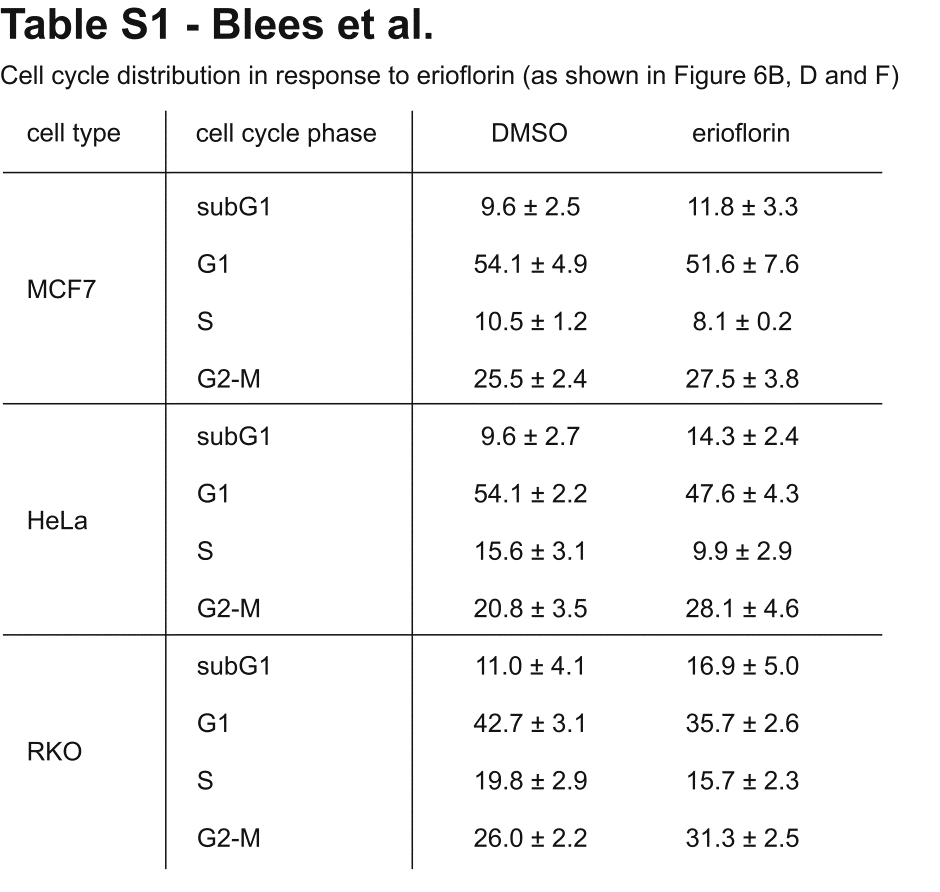
**

Supplement: Table S1 — Cell cycle distribution in response to erioflorin. (DOC) [file pone.0046567.s005.doc]
